# Spirit of the Foreign Periodicals, &c.

**Published:** 1839

**Authors:** 


### Spirit of the Foreign Periodicals, &c.

### On Re-vaccination.

M. Dezeimeris has published an excellent paper in the *Experience*, in continuation of his former one—which we alluded to with praise in our article on Re-vaccination in the Foreign Periscope of our last Number. We shall again avail ourselves of his researches on the same subject, as it is one which, at the present time especially, requires to be well understood by all medical men.

M. *Dezeimeris* endeavours to establish the following positions.

1. That the cases of small-pox occurring in vaccinated persons have of late years become progressively more and more numerous during each successive epidemic.

2. That the severity of the small-pox in vaccinated persons has gradually increased with the number of the persons so affected.

3. That vaccinated persons become more and more liable to be affected with small-pox as they advance in life; and that the severity of the disease in them is, on the whole, proportionate to the interval of time that has elapsed since the operation was performed.

4. That this law fixes the degrees of susceptibility of the system to the vaccine virus as well as to the variolous contagion; and that *it* thus regulates the chances of successful re-vaccination and of the liability to small-pox.

5. That re-vaccination is as effectual a preservative to those in whom the protecting influence of the first vaccination may have passed away, as this first operation proved to be during several years.

The *first* of these propositions was very fully illustrated in the last Number of this Review (Rev.), by reference to the statistical data from various countries of Europe. It is perhaps scarcely necessary to adduce any further examples of its truth; but as we did not allude to any reports from Italy, we shall now very briefly supply that omission.

Dr. *Verson*, physician of the circle of Parenzo in Istria, informs us that, from 1802 to 1819, the cases of small-pox after vaccination were so rare that little credit was attached to them; but that from the latter year to 1825 the increase of such cases was so considerable and progressive, that no one could remain sceptical as to the insufficiency of the protection. In 1832-33, the number of cases of variolous and varioloid affections observed by Dr. V. was 300, the greater number of which occurred in vaccinated persons. All the severe cases occurred in persons from the age of 18 to that of 35 years. Dr. *Verson* is a strenuous advocate of re-vaccination.

Dr. *Gambarini*, physician of the Milan Hospital, mentions that the number of cases of small-pox after vaccination was considerably greater in the epidemic of 1830 than in that of 1825, and that, previously to the latter year, such cases had been of very rare occurrence.

These facts, coupled with the numerous others reported from different parts of Italy, and with those which have been observed in other countries, leave no doubt as to the fact that the frequency and severity of variolous epidemics have much increased, in almost every part of Europe, within the last twelve years.

The *second* position—viz. that the severity of the variolous and varioloid epidemics has certainly been increasing of late years—is fully borne out by the various histories which have been laid before the public. During the first ten or even twenty years after the general introduction of vaccination, the cases of small-pox occurring in persons who had been vaccinated were not only very rare, but almost invariably very mild and much modified too in their characters. The primary fever subsided when the eruption appeared; most of the papulæ dried off without suppurating, and the others maturated in the course of a few days: a fatal case was seldom or never heard of in these days.

The disease however in subsequent epidemics has been gradually approaching more and more nearly to the *natural* disease, such as occurs in persons who have not been either vaccinated or inoculated; and in some recent epidemics, the cases which have occurred after vaccination have been in no respect different from those of the *natural* disease. According to Dr. *Grancini*, the mortality in such cases has of late years been about 7½ per cent.

The *third* position may be equally satisfactorily established. If we consult Dr. *Heim* on the cases of secondary variola observed at Wurtemburg during the last six years, we cannot fail to remark with what efficacy vaccination protects the system for ten or twelve years, after it has been properly performed; and that it is only after that period that its preservative effects manifestly become less decided—the degree or extent of decrease in the protecting power seeming to be strictly proportionate to the length of the interval that has elapsed since the virus had been inserted.

Whereas 216 cases of variola were observed by him in *unvaccinated* children under ten years of age, and only 51 in *vaccinated* children within the same period of life, there were 71 cases of the disease in unvaccinated persons of from 11 to 20 years of age, and not fewer than 507 cases in vaccinated persons of from 13 to 24 years of age.*

Dr. *Bidder*, medical inspector of *Courland*, informs us that, from January 1826 to the end of 1831, out of 618 cases of variolous and varioloid disease—the greater number in vaccinated persons—observed by him, and of which 94 proved fatal, every one occurred at an interval of from 10 to 20 years from the date of the preceding vaccination.

Dr. *Nadherney*, first physician of *Bohemia*, states that, during the year 1828, variolous disease was very extensively diffused in that country. All the cases, which were observed in vaccinated persons, were found upon enquiry to have occurred at an interval of from 12 to 24 years after the date of the vaccination.

Dr. *Pommer*, in his report on the sanitary state of the Canton of *Zurich*, has recorded numerous facts in confirmation of the same truth. Persons, who had been vaccinated from 8 to 15 years before the epidemic, were affected very mildly; but, after the latter age, the disease was almost always much more severe, and approximated more in its characters to the *natural variola*.

A very severe variolous epidemic prevailed at *Vienna* in 1832. Dr. *Gunther*, physician of the Orphan Hospital in that city, mentions as a proof of the protecting power of vaccination, that in the establishment under his care, which contains upwards of 1300 children—the greater number of whom were under 10 years of age—eleven only were affected by the contagion. The master and mistress of the hospital suffered severely.

It is almost unnecessary to multiply examples of the position—that the severity of small-pox occurring after vaccination is almost invariably proportionate to the length of the interval that may have elapsed since the date of the operation.

We might easily adduce other proofs of its truth from the writings of Dr. *Festler of Padua*, of Dr. *Gambarini of Milan*, &c.; but, as we cannot afford room for such details, we shall now proceed to the proofs of the *fourth*

* To explain this seemingly disproportionate excess of the cases in vaccinated over those in unvaccinated patients, we must remember that vaccination is made obligatory by the government of Wurtemburg, and hence that few persons evade it.

position—viz. that the susceptibility of the system to be affected either by the variolous or by the vaccine virus after vaccination is inconsiderable for twelve or fifteen years, but becomes more and more decided after the lapse of that period.

Dr. *Reuss* of *Stuttgard* mentions that out of 27 re-vaccinations, of which 25 were performed on persons under 14 years of age, three only took effect; whereas 7 out of 12 re-vaccinations on persons above that age succeeded.

Dr. *Lucas* of *Erkelenz* performed, in 1833, 289 re-vaccinations on persons from 2 to 28 years of age. The operation was followed by only an insignificant irritation in children under five years of age; by imperfectly formed vesicles in those under ten years; and by more or less complete and normal vaccine vesicles in those above that period of life. Almost uniformly he found that the success of the operation was proportionate to the length of interval that had elapsed since the date of the former one.

He tried also the effects of vaccinating some persons who had passed through the natural small-pox. The pustules or vesicles which followed were always observed to be more perfect in those persons, who exhibited but few pits or scars of the small-pox. In two persons, both of whom were upwards of forty years of age, and who had had small-pox in their childhood, vaccination was followed by the most perfect cow-pock vesicles.

In 1830, Dr. *Pini* of *Morbegno* re-vaccinated thirty-five persons of various ages from 4 to 20 years, and in all of whom there were good cicatrices of a former vaccination on their arms. In the children from 4 to 10 years of age, the punctures were followed only by the most trifling local irritation, which quite passed away in thirty-six hours. In the others, vesicles, which were observed to be more or less complete and normal just in proportion to the more advanced ages of the patients, were formed at the seat of the punctures. In some, these vesicles exhibited all the appearances of genuine primary vaccine vesicles. Not one of these re-vaccinated young people contracted the variolous epidemic, which was very prevalent in the district at the time.

Dr. *Baumgartner* of *Baden*, in a recent epidemic of variola there, which attacked many persons who had been vaccinated and proved fatal in a good many instances, strongly recommended that all persons above 12 years of age should be immediately re-vaccinated. Not a single case of small-pox was afterwards observed in any one who had been so treated.

At *Riegel*, of 910 persons, who were re-vaccinated, 161 exhibited genuine cow-pock vesicles after the operation.

But it is needless to cite more proofs: we shall only mention the names of the veteran Dr. *Wagner*, of Schlieben, Dr. *Pommer*, of Zurich, Dr. *Balardini*, of Valteline, Dr. *Festler* of Padua, &c., all of whom have of late years practised re-vaccination, and continue to recommend the practice as the most effectual means of arresting the spread of variolous contagion.—*L'Experience.*

